# Suffering a Healthy Life—On the Existential Dimension of Health

**DOI:** 10.3389/fpsyg.2022.803792

**Published:** 2022-01-27

**Authors:** Per-Einar Binder

**Affiliations:** Department of Clinical Psychology, Faculty of Psychology, University of Bergen, Bergen, Norway

**Keywords:** existential health, existential concerns, definitions of health, medicalization of life, suffering

## Abstract

This paper examines the existential context of physical and mental health. Hans Georg Gadamer and The World Health Organization’s conceptualizations are discussed, and current medicalized and idealized views on health are critically examined. The existential dimension of health is explored in the light of theories of selfhood consisting of different parts, Irvin Yalom’s approach to “ultimate concerns” and Martin Heidegger’s conceptualization of “existentials.” We often become aware of health as an existential concern during times of illness, and health and illness can co-exist. The paper discusses how existential suffering in Western culture is described, to an increasing degree, as disorders or psychological deficits, and perfectionistic health goals easily can become a problem. We seek to avoid suffering rather than relate to it, with all the tension that may create. The paper argues that suffering is an unavoidable aspect of people’s experience of their lives, and actively relating to suffering must be regarded as a fundamental aspect of health. The need and usefulness of a concept of “existential health” is discussed.

## Introduction

Health is usually what we expect, and it is certainly not a problem. Therefore, health is not necessarily something we reflect about when it is present, and it is often a silent and unnoticed phenomenon (Gadamer, 2018). When we try to conceptualize what health is, a negative definition—absence of illness—is often what comes most immediately to mind. Our need for reflection and story-making arises when we are in trouble, when we have a problem to solve, or something unexpected occurs, such as when illness shows up (Kirmayer, 2000).

Mostly, we become aware of the existential dimension of health during times of illness. When we are ill, we more easily become aware of the finite nature of our being-in-the-world (Yalom, 1980). Illnesses might bring limitations to the activities in life that engage us and give direction. Often illnesses heighten our awareness of our mortality (Kissane, 2012). The uncertainty connected with illness also may also confront us with the undetermined nature of our existence. It challenges us with the fact that the choices we have made so far do not constitute an essence of who we are. Often, our sense of freedom and responsibility feels troublesome to us. This can be mixed with sorrow when we use our freedom to let go of some aspect of our lives. However, sometimes it also opens up the freedom to choose a topic and style for a new chapter in one’s life story, not seldom with an unexpected twist (McAdams and Bowman, 2001). A disease can make us reconsider our projects and roles, and demand that we make new choices and priorities. And this process, can be healthy. Illness and a healthy, heightened, and existential awareness can co-exist.

The aim of this paper is to examine the existential context of physical and mental health. The definitions of health made by Hans-Georg Gadamer (2018) and the World Health Organization will be used as a starting point. They will be discussed in light of perspectives on suffering (Schneider, 1999; Miller, 2005), and selfhood consisting of different parts (Bromberg, 1996). Then I will examine the relationship between health and existential concerns through the approaches of Irvin Yalom (1980) and Martin Heidegger (1957), and Ola Sigurdson’s (2016, 2019) definition of “existential health.” Medicalized and perfectionistic ideals for health are critically discussed, considering the existential conceptualizations. I will propose that being aware of suffering and actively relating to it is part of living a healthy life, and I will also discuss whether a concept of existential health is needed.

## What Is Health?

When we are healthy, other aspects of life than health in itself seems more important. We are busy living. Gadamer (2018) describes this as “the enigma of health”: our health is of uttermost importance to us, but at the same time, it is not something we usually reflect upon or examine through introspection. When we are healthy, we are in a “condition of being involved, of being in the world, of being together with one’s fellow human beings, of active and rewarding engagement in one’s everyday tasks” (p. 13).

When we are engaged in our everyday world in a relatively undisturbed way, we feel that this world has what Heidegger (1957) describes as a “homely” quality. Fredrik Svenaeus (2019) explains that illness might transform our experience of everyday life and get with it an “unhomelike” being-in-the-world. Illness might feel like something unfamiliar or alien intruding, either in the body or the mind. Illnesses quickly also penetrate much of our emotional lives. It requires us to change how we imagine the near and sometimes far future. Also, not bringing attention to illness takes effort. As Svenaeus points out, diseases may make us even more preoccupied with going on with our everyday life matters, simply because these matters become hard and painful to accomplish. We find parallels to this line of thinking in Joseph Sandler’s (Sandler, 1960) concept of the “background of safety” as a contrast to the world of the traumatized person, where the world becomes a “background of the uncanny.” Health is part of our sense of homeliness and safety in the world.

Gadamer (2018) also describes “well-being” as an aspect of health. According to Gadamer, this type of well-being, of greatest value to our lives, mostly escapes our awareness. This well-being is not something that draws our attention in itself, because it is a condition “of being unhindered, of being ready for and open to everything?” (p. 73). But might health as well be more than a pre-reflective phenomenon, working in the background when we are busy living our lives?

The World Health Organization goes some steps further to get a positive conceptualization of health. It defines it as a “a state of complete physical, mental and social well-being and not merely the absence of disease and infirmity.” The use of the word “complete” may be interpreted in different ways. On the one hand, the WHO infer a balance of attention paid to overall health’s physical, mental, and social parts. On the other hand, the word “complete” can allure to see health as an ideal state, out of reach for most of us, perhaps except in the luckiest and happiest moments of our lives.

Paradoxically, one can argued that “complete” well-being in an idealized sense of the word would make us sick in the long run. Our immune system as well as our musculoskeletal system and our emotional and cognitive abilities are strengthened and keep functioning through stresses and challenges. You need to catch a cold sometimes to keep your immune system strong. People who experience meaning in their life are also often the people who experience a certain amount of stress and worry (Baumeister et al., 2013). Health is not a thing, or something that we possess, but a way of living. This is also something the WHO has taken into account, and in 1986, the WHO also expanded health to encompass “resources for everyday life.” In this way, the organization emphasized resilience and the active part of health through dealing with life’s challenges.

When Gadamer (2018) describes health as a condition of both being unhindered, and ready for and open to everything, this is also certainly much of an ideal state. Life is full of hindrances, there is often a lot going on that we are not especially ready for, and certainly we often close down rather than open up. We experience huge contrasts in life. Therefore, it can be argued, the experience of being unhindered, ready, and open is something we often notice, even reflect on. These are moments when we feel vitality, and often joy. We might feel grateful for our joys because we are aware of the place suffering has in our life as a whole (Vaillant, 2008). Perhaps health has more to do with the ability to be present in the contrasts of life? Furthermore, perhaps health also has to do with our ability to handle suffering, as an unavoidable fact of life?

The title of Jon Kabat-Zinn and Hanh’s (2009) book *Full catastrophe living* signals that all lives also have a “catastrophic side,” and that it is possible to stay present in this mix of catastrophe and suffering, and joy and growth that a human life offers. Anger, anxiety, shame, guilt, envy, and sorrow are messengers of bad news. These emotional states are not a trick evolution has made to bother us; they are messengers about realities in our life that we need to address. We suffer when we experience something hindering us, when we feel lonely, or that we lack something, when we feel rejected or threatened, and when we have lost something of great importance to us. Negative emotions have an important place in a rich and meaningful life (Parrott, 2014).

When something is unpleasant and painful, in current culture we often go to the language of illness, and seek explanations in terms of dysregulation or disorders. As Kirk Schneider (1999) points out, when “dysregulation of the emotional brain” (p. 109) is to be described quite unambiguously in mechanical and quantitative terms, suffering is a multifaceted problem relating to dilemmas in existence itself. Suffering entails an alteration, and sometimes a rupture in our life – it is a messenger of change. Illnesses sometimes cause such a rupture, and suffering can be strongly related to a disorder. Important knowledge of high relevance to treatment might be systemized through diagnostic terms. Although this knowledge can be helpful, it also brings a danger of changing our language for the painful challenges in life. When disorders, understood in diagnostic terms, are something to eliminate, suffering is something to deal with. This is also a central part of healing processes. As Ronald B. Miller (2005) points out, psychotherapy primarily deals with suffering. Surprisingly, this is often an unaddressed fact. In an increasing degree, clinical psychology and psychotherapy has been put under pressure by insurance companies and national health services to adapt to the diagnostic language of disorders when making a treatment plan. When it comes to the conceptualization of the patient’s problem, clinical psychology and psychotherapy lose something crucial if the medical vocabulary of “disorders” comes in the way of relating to human suffering in an existential sense. Diagnosed “disorders” are abstractions at a considerable distance from the personal life of meaning and importance. Suffering is something humans have in common, and therefore it can be shared. Patients’ experience of therapists’ empathy is ranked among the most important factors of change in psychotherapy (Norcross, 2011). When psychotherapy works, shared experience of suffering is a fact. The therapist can sense some of the patient’s pain, both in their imagination and their body. Moreover, the patient becomes able to relate to their own suffering through the recognition and compassion offered by another human being. There are paths to health that do not have to do with “elimination of illness,” but by relating to suffering in friendly, caring, and accepting ways, both in others and in oneself (Esdaile et al., 2021).

Based on a theory of selfhood consisting of multiple parts, Phillip Bromberg (1996) states that “health is the ability to stand in the spaces between realities without losing any of them—the capacity to feel like one self while being many” (p. 166). This need to relate to different parts of self, while being a whole person at the same time, is also in line with what Donald Winnicott (2014) described when he stated that “we are poor indeed if we are only sane” (p. 150).

George Vaillant (2008) describes how joy allows us acknowledge and relate to suffering. A part of my self might feel sorrow over a loss, another part is engaged in a very meaningful project. Both parts are authentic and true, and the sum is bittersweet. I might suffer from a chronic and sometimes painful disease, and at the same time fall in love, and feel that life is opening up deeply; one part of me is in pain, another is on the wings of love. If I can stay present with such contrasts, and they are allowed to be part of me and my life—if I can handle them and feel agency—perhaps this is the highest possible level of health that I can reach? To illuminate this question, we need to examine health as an existential phenomenon.

## Health and Existential Concerns

Irvin Yalom (1980) describes four major “ultimate concerns”: death, meaninglessness, isolation, and freedom. He describes these as “givens of existence,” or an “inescapable part” of being human, and that every person must come to terms with these concerns through active choices to realize their individual potential. These ultimate concerns have common roots with what Heidegger (1957) describes as “existentials,” which are structures that form human experience, and that are rooted in our ontological being. The fundamental basis in this structure is “Caring” (*Sorge*), a quality of engagement in the world. “Understanding” (*Verstehen*), “Being-with” (*Mit-Sein*), “Being-toward-death” (*Sein-zum-Tode*), and “Mood” (*Befindtlichkeit*) are other examples of existentials. In our discussion of the existential dimension of health, Yalom’s description of the psychological and phenomenological aspects of “ultimate concerns” might be more directly relevant than the more wide-ranging concepts on an ontological level.

However, a problem with Yalom’s (1980) conceptualization of ultimate concerns is that they highlight only one of two polarities. He intends to explore a polarity by addressing the pole we tend to resist. For example, when he describes meaninglessness as an existential concern, he uses this as a point of entry to explore the role of meaning-making. Creating and discovering meaning in one’s own personal way is necessary to live in an authentic and fulfilling way. As Heidegger (1957) points out, in our everyday mode of experiencing the world, we experience the world as a place where meaning already exists. We are involved in our everyday goal-related tasks in a way that makes us understand them (cf. Heidegger’s term “Understanding”). However, existential anxiety is a mood that disrupts our involvement with the familiar signifiers of the world, wipes away the intelligibility we take for given. In this way it also confronts us with meaninglessness. In this way, existential anxiety calls for us to reorient ourselves and commit ourselves to engagements that create new meaning. A possible modification of Yalom’s concepts that might be useful in our discussion is to describe these existential concerns as polarities:

(1) Death—and awareness of living a life of one’s own.
(2) Meaning—and meaninglessness.
(3) Being-with—and isolation.
(4) Freedom—and limitations and conditionings.

Discussing the existential dimension of health, “embodiment and emotional being” is relevant as a fifth concern; health becomes an issue for us since we are bodily beings. Neither Yalom nor Heidegger explicitly discusses this, however, Heidegger signals that an emotional aspect, “Mood” *(Befindtlichkeit)*, is also a way of being that gives us access to our world. Maurice Merleau-Ponty (1962) more actively addresses the fact that our-being-in-the-world is embodied, and Eugene Gendlin (1982) discusses how this embodied mode of being also produces a felt sense of emotional meaning in us. We can be immersed in our bodily felt experience and witness and reflect on these experiences through our capacity for awareness. Our embodied and emotional being has both a proactive and receptive side. Strength and agency on the one side, and vulnerability and receptivity on the other, are polarities connected to embodiment as an existential concern.

Both mental and physical health involves our existential concerns. Our worries about health provide a clue to what types of concerns are involved. Worries about physical illness are often triggered by death-anxiety and the fact that we are embodied beings (Solomon et al., 2015). The strange-looking mole, the uneven heartbeat, the inexplicable tiredness—they easily trigger inner scenarios with death as a possible outcome, and sometimes not without reason. Worries about physical illness, as well as mental illness, can also be connected to concerns about loss of freedom, isolation, and meaninglessness. Worries can both be an invitation to constructive action, and a source of avoidance and passivity (Sweeny and Dooley, 2017). After we have taken the relevant action, death, the possibility of unfreedom, isolation, and meaninglessness are still a part of our lives, and we are still living in a vulnerable and sometimes emotionally sensitive body. In even the healthiest of lives, suffering is lurking around the corner.

If we are to paraphrase Bromberg (1996), we might say that health depends on the ability to “stand in the spaces” (p. 166) between existential polarities:

(1) We can relate to the fact that we will die, and use this insight in a way that enriches our being (Heidegger, 1957); for instance, insight into this fragility of being can invite us to make life choices in line with what we experience to be most important in life (Jaspers, 2010).
(2) We cannot extract meaning from the universe in itself. Meaning is something we create, collectively through language and culture, and individually, through our everyday engagements with other human beings and goal-related tasks with objects in our world (Heidegger, 1957). Through works of art, creativity, and play, we transform our experience of the world and find new meaning (Winnicott, 1971; Heidegger, 2000). Through engagements in activities and projects that we value and that point to something larger than ourselves, we create meaning in our lives (Frankl, 2004). Since meaning depends on our own efforts, meaninglessness and lack of purpose are always a threat becoming apparent through ruptures in our everyday engagements and existential anxiety (Tillich, 2000).
(3) We can relate to the fact that we live lives deeply connected to the destiny of other people and that life is fundamentally relational (Heidegger, 1957; Buber, 2003). At the same time parts of ourselves and our lived experience is out of reach from others, and therefore isolated (Winnicott, 1965). We also recognize that there are parts of other persons’ lived experience that we never can directly know. Both being-with and isolation are parts of our lives.
(4) We have a fundamental sense of freedom and responsibility (May, 1999). At the same time, we can relate to the fact that we are limited by habitual ways of being that we are often only dimly aware or unaware of, and that social structures and power limit what choices we see as possible. In all lives, people sometimes must struggle to make the choices they want and need to take.
(5) We can experience ourselves in a body that is felt to be our own, and sensing this body make us feel grounded (Winnicott, 1954). All types of human experience involve a touch-like sense of belonging, also when the content of our feelings are not about the body, but about events in the world that we are part of Ratcliffe (2009). Through the body, we can relate to sensations and emotional states that tell us about what is going on in our world, both at the physical and relational level (Gendlin, 1992). Both agency and receptivity are essential aspects of our bodily involvement (Merleau-Ponty, 1962). This embodied being-in-the-world implies strength and vulnerability as polarities in ourselves, sometimes playing together, sometimes in conflict with each other.

When it comes to health, being-with might be regarded as the most important point of entry, also to be able to reach the other existential dimensions of health. As Daniel Siegel (2010) points out, “feeling felt” is a precondition for psychological growth, especially critical for attachment in infancy, but also for developmental processes later in life. “Feeling felt” describes the ability of one person to encounter another person empathically and authentically on an emotional level. When feeling felt, we intuitively grasp that this other emotionally recognizes our emotional experiences. We feel the emotional resonance. When the others communicate how they perceive us as experiencing persons, this builds and strengthens our sense of existing and being alive. Feeling felt creates a fundamental sense of safety. This emotional bond is a primary mode of being-with, and of utmost importance for being able to handle suffering in life, when we need to create meaning, make choices, when we struggle to be grounded in our bodies, and when we face death.

## Conceptualizations of Existential Health

Does this way of understanding the existential dimensions of health imply that we need a distinct concept of “existential health”? Several authors have proposed this.

One way of conceptualizing existential health places it as a specific dimension of health in addition to other dimensions. Valerie DeMarinis (2008) describes this existential dimension as a focus on the individual’s understanding of existentiality and the way he or she creates meaning. Her concept of existential health is closely linked to participation in meaning-making systems, including those of spirituality and religion, and the ways of relating to rituals and symbolic expressions that are part of such systems. Melder (2012) develops a similar conceptualization, describing existential health as a distinct sphere in addition to the physical, mental, social and ecological, and elaborates on the psychological and creative dimension. She uses Winnicott’s theory of the root of creativity in childhood playfulness to explore how the individual projects existential needs into the world, and makes interpretations of it, introjecting from experiences of factors in the outer world that have existential significance, such as religions and philosophies. In her view, the existential health sphere is the sum of such existential meaning-making processes.

Sigurdson (2016) proposes another way to understand existential health. He suggests “existential health” to be the reflexive experience of health. This experience has an intentional quality in how the person relates to their ailment and health. The experience also must have a personal quality; the person relates to this experience as theirs. In this way, he distinguishes between existential health and concepts that relate to more specific dimensions of health, such as spiritual health, and physical, mental, and social well-being. Although his conceptualization mainly addresses the reflexive dimension, Sigurdson (2019) also discusses how embodiment and the way we relate to suffering is a central aspect of existential health. He proposes that we achieve health through learning how to suffer. Although suffering can be seen as a result of our vulnerability as bodily and emotional beings, Sigurdson does not regard it as a passive sensation. Suffering is an active work of acknowledging pain or misfortune, “establishing a kind of solidarity, and learning how to endure it” (p. 96). Suffering means to take an intentional and proactive stance toward painful psychological or bodily realities rather than passively reacting to them. In this sense, the act of suffering and existential health is essentially one and the same.

When DeMarinis and Melder puts existential health closer to the spiritual dimension of health, Sigurdson describes it as a more overarching concept, relating to all aspects of health. The way he connects existential health to embodiment and the ability to “suffer” make his conceptualization more in line with the discussion in this paper. I would add that we need a concept of being simply “aware” in addition to being “reflexive” when it comes to the existential dimension of health, as our relationship to health does not have to be discursive. Our state of being-in-the-world can be also recognized in a more “silent,” open, receptive, and at the same time friendly way, as in mindfulness-meditation (Kornfield, 2017). However, in the context of this discussion, this is not the most important distinction.

Do we need existential health in addition to other conceptualizations, or are we more in need of an existential reexamination of the meaning of “health” and its place in life? This does not have to be an either/or, but in a society where health has turned out to be a life goal on its own, this is an important issue to discuss.

## The Medicalization of Existential Realities

Medical terms are creeping more and more into our everyday language. Experiences of sorrow easily becomes “depression,” overwhelming experiences becomes “trauma,” fear of failure becomes “anxiety.” Concepts from psychology have also crept into our everyday language and the way we handle our life (Madsen, 2014). For instance, we are a culture that seem obsessed with the idea of “self-esteem,” tending to see the experience of self-worth as decontextualized from our real efforts and social engagements (Baumeister et al., 2003). Self-esteem has become something we think we possess inside ourselves, which we need to build, and fear losing.

On the one side, our existential suffering, to an increasing degree, is in danger of being described as disorders or psychological deficits (Albarracin et al., 2015). On the other side, one can also argue that there is a cultural trend where we can easily be invited to pursue quite perfectionistic health goals (Fugelli, 2006; Sirois, 2016). In medicine, such health goals may lead more people to be “patients,” whereby they receive treatment for potential risks or plastic surgery for normal variants of bodily appearance (Suissa, 2008; Brownlee et al., 2017).

As our lives become medicalized in this way, there is also a danger that we become foreign to them (Miller, 2015). Our troublesome mental states may become less familiar to us, and become the domain of specialists in clinical psychology and medicine. Instead of reflecting our life, our personal struggles, and our values, mental states then gradually came to belong to the categories of health and unhealth. As Patrick Whitehead (2019) points out in his book *Existential health psychology*, treatment always transforms us as people—a bit of us becomes medicalized.

In the strict medical sense, good health is in itself highly compatible with existential health. As the second half of life gives rise to several challenges to both physical and mental health and existential struggles, it is interesting to see how existential health both might be a buffer against illness and bolster and build health also in these domains. Experiencing mastery and purpose in life, being engaged in something bigger than oneself, and cultivating positive social relationships have been shown to predict better self-rated health, less disability, healthier profiles of biological risk, greater well-being, and better cognitive function in aging adults, even in the context of disability and chronic illness (Ryff et al., 2012). A sense of purpose in life is associated with a reduced risk for all-cause mortality (Cohen et al., 2016). And the value of *not* becoming foreign to negative emotions and being able to relate to a broad diversity in feelings and mental states is also of great importance to mental as well as physical health (Parrott, 2014; Quoidbach et al., 2014). Therefore, health promotion policies also should include such perspectives. However, there is a paradox at play if purpose, meaning, and our capacity to relate to suffering become means with health as a goal. This paradox parallels the one that Yalom (2012) describes when he says that although we need meaning in life, and lack of meaning is deeply disturbing for us, it is something that we are less likely to find the more we deliberately pursue it. Meaning ensues from meaningful activity; meaningfulness is a byproduct of engagement and commitment. Similarly, a good health outcome is more like a bonus we gain from our willingness to face existential challenges; it is not the main reason we engage in them in the first place. Illness will always sooner or later hit us. A concept of existential health might highlight some important possibilities in life: In an existential sense you might die healthy, in a medical sense none of us do.

## Conclusion

We might become healthier through our existential struggles; meaning is essential for a healthy life. But at the deepest level, we do not face our existential challenges to become healthy. We struggle with them because purpose, meaning, and existential concerns are of the highest value for our lives. In a highly medicalized society, we need to reinvent our language for these existential struggles, and the suffering that always accompanies them. If a concept of “existential health” can make us more aware of the fact that the existential concerns are always part of both health and illness, it is worth developing.

## Data Availability Statement

The original contributions presented in the study are included in the article/supplementary material, further inquiries can be directed to the corresponding author.

## Author Contributions

The author confirms being the sole contributor of this work and has approved it for publication.

## Conflict of Interest

The author declares that the research was conducted in the absence of any commercial or financial relationships that could be construed as a potential conflict of interest.

## Publisher’s Note

All claims expressed in this article are solely those of the authors and do not necessarily represent those of their affiliated organizations, or those of the publisher, the editors and the reviewers. Any product that may be evaluated in this article, or claim that may be made by its manufacturer, is not guaranteed or endorsed by the publisher.

## References

[B1] Albarracin D. Ducousso-Lacaze A. Cohen D. Gonon F. Keller P.-H. Minard M. (2015). There is no cure for existence: on the medicalization of psychological distress. *Ethic. Hum. Psychol. Psychiatry* 17 149–158.

[B2] Baumeister R. F. Campbell J. D. Krueger J. I. Vohs K. D. (2003). Does high self-esteem cause better performance, interpersonal success, happiness, or healthier lifestyles? *Psychol. Sci. Public Inter.* 4 1–44. 10.1111/1529-1006.01431 26151640

[B3] Baumeister R. F. Vohs K. D. Aaker J. L. Garbinsky E. N. (2013). Some key differences between a happy life and a meaningful life. *J. Posit. Psychol.* 8 505–516.

[B4] Bromberg P. M. (1996). Standing in the spaces: the multiplicity of self and the psychoanalytic relationship. *Contemp. Psychoanal.* 32 509–535. 10.1080/00107530.1996.10746334

[B5] Brownlee S. Chalkidou K. Doust J. Elshaug A. G. Glasziou P. Heath I. (2017). Evidence for overuse of medical services around the world. *Lancet* 390 156–168. 10.1016/s0140-6736(16)32585-5 28077234 PMC5708862

[B6] Buber M. (2003). *Between Man and Man.* London: Routledge.

[B7] Cohen R. Bavishi C. Rozanski A. (2016). Purpose in life and its relationship to all-cause mortality and cardiovascular events: a meta-analysis. *Psychosom. Med.* 78 122–133. 10.1097/PSY.0000000000000274 26630073

[B8] DeMarinis V. (2008). The impact of postmodernization on existential health in Sweden: psychology of religion’s function in existential public health analysis. *Arch. Psychol. Relig.* 30 57–74. 10.1163/157361208x316962

[B9] Esdaile A. S. Shah F. Binder P.-E. (2021). *Eksistensiell Helse er Blitt et Nøkkelbegrep Under Pandemien [Existential Health Has Become a Key Concept in the Pandemic].* Psykologisk.no.

[B10] Frankl V. E. (2004). *Man’s Search for Meaning: The Classic Tribute to Hope From the Holocaust.* New York, NY: Random House.

[B11] Fugelli P. (2006). The Zero-vision: potential side effects of communicating health perfection and zero risk. *Patient Educ. Counsel.* 60 267–271. 10.1016/j.pec.2005.11.002 16469471

[B12] Gadamer H.-G. (2018). *The Enigma of Health: The Art of Healing in a Scientific Age.* Hoboken, NJ: John Wiley & Sons.

[B13] Gendlin E. T. (1982). *Focusing.* New York: Bantam.

[B14] Gendlin E. T. (1992). The primacy of the body, not the primacy of perception. *Man and World* 25 341–353.

[B15] Heidegger M. (1957). *Zein und Zeit [Being and Time].* Tübingen: Niemeier.

[B16] Heidegger M. (2000). *Kunstverkets Opprinnelse, Med en Innføring av Hans-Georg Gadamer [The Origin of the Work of Art, With an Introduction by Hans-Georg Gadamer].* Oslo: Pax.

[B17] Jaspers K. (2010). *Philosophy of Existence.* Philadelphia, ON: University of Pennsylvania Press.

[B18] Kabat-Zinn J. Hanh T. N. (2009). Full Catastrophe Living: Using the Wisdom of Your Body and Mind to Face Stress, Pain, and Illness. New York, NY: Delta Trade.

[B19] Kirmayer L. J. (2000). “Broken narratives: clinical encounters and the poetics of illness experience,” in *Narrative and the Cultural Construction of Illness and Healing*, eds Mattingly C. Garro L. C. (Berkeley,CA: University of California Press), 153–180.

[B20] Kissane D. W. (2012). The relief of existential suffering. *Arch. Int. Med.* 172 1501–1505. 10.1001/archinternmed.2012.3633 22945389

[B21] Kornfield J. (2017). *No Time Like the Present: Finding Freedom, Love, and Joy Right Where You Are.* New York, NY: Simon and Schuster.

[B22] Madsen O. J. (2014). *The Therapeutic Turn: How Psychology Altered Western Culture.* Abingdon: Routledge.

[B23] May R. (1999). *Freedom and Destiny.* New York, NY: WW Norton & Company.

[B24] McAdams D. P. Bowman P. J. (2001). “Narrating life’s turning points: redemption and contamination,” in *Turns in the Road: Narrative Studies of Lives in Transition*, eds McAdams D. P. Josselson R. Lieblich A. Washington, DC: American Psychological Association, 3–34.

[B25] Melder C. (2012). The epidemiology of lost meaning: a study in the psychology of religion and existential public health. *Scrip. Instit. Donner. Aboen.* 24 237–258. 10.30674/scripta.67417

[B26] Merleau-Ponty M. (1962). *Phenomenology of Perception.* London: Routledge.

[B27] Miller R. B. (2005). Suffering in psychology: the demoralization of psychotherapeutic practice. *J. Psychother. Integrat.* 15 299–336.

[B28] Miller R. B. (2015). *Not so Abnormal Psychology: A Pragmatic View of Mental Illness.* Washington, DC: American Psychological Association.

[B29] Norcross J. C. (2011). *Psychotherapy Relationships That Work: Evidence-Based Responsiveness.* Oxford: Oxford University Press.

[B30] Parrott W. G. (2014). *The Positive Side of Negative Emotions.* New York, NY: Guilford Publications.

[B31] Quoidbach J. Gruber J. Mikolajczak M. Kogan A. Kotsou I. Norton M. I. (2014). Emodiversity and the emotional ecosystem. *J. Exp. Psychol. Gen.* 143:2057. 10.1037/a0038025 25285428

[B32] Ratcliffe M. (2009). Existential feeling and psychopathology. *Philos. Psychiatry Psychol.* 16 179–194.

[B33] Ryff C. Friedman E. Fuller-Rowell T. Love G. Miyamoto Y. Morozink J. (2012). Varieties of resilience in MIDUS. *Soc. Pers. Psychol. Comp.* 6 792–806. 10.1111/j.1751-9004.2012.00462.x 24058379 PMC3775270

[B34] Sandler J. (1960). The background of safety. *Int. J. Psycho-Anal.* 41 352–356. 13746183

[B35] Schneider K. J. (1999). Suffering and its ambiguities. *Psychother. Pat.* 11 109–114. 10.4324/9781315786360-8

[B36] Siegel D. J. (2010). *Mindsight: The New Science of Personal Transformation.* London: Bantam.

[B37] Sigurdson O. (2016). Existential Health. Philosophical and historical perspectives. *LIR J.* 6 8–26.

[B38] Sigurdson O. (2019). “Only vulnerable creatures suffer: on suffering, embodiment and existential health”, in *Phenomenology of the Broken Body*, eds Dahl E. Falke C. Eriksen T. E. (New York, NY: Routledge), 87–100.

[B39] Sirois F. M. (2016). *“Perfectionism and health behaviors: a self-regulation resource perspective,” in *Perfectionism, Health, and Well-Being**, eds Sirois F. M. Molnar D. S. (Berlin: Springer), 45–67.

[B40] Solomon S. Greenberg J. Pyszczynski T. (2015). *The Worm at the Core: On the Role of Death in Life.* New York, NY: Random House.

[B41] Suissa A. J. (2008). Addiction to cosmetic surgery: representations and medicalization of the body. *Int. J. Ment. Health Addict.* 6 619–630. 10.1007/s11469-008-9164-2

[B42] Svenaeus F. (2019). A defense of the phenomenological account of health and illness. *J. Med. Philos. Forum Bioeth. Philos. Med.* 44 459–478. 10.1093/jmp/jhz013 31356662 PMC6663122

[B43] Sweeny K. Dooley M. D. (2017). The surprising upsides of worry. *Soc. Pers. Psychol. Comp.* 11:e12311. 10.1111/spc3.12311

[B44] Tillich P. (2000). *The Courage to Be.* London: Yale University Press.

[B45] Vaillant G. (2008). *Spiritual Evolution: A Scientific Defense of Faith.* New York, NY: Harmony.

[B46] Whitehead P. M. (2019). *Existential Health Psychology: The Blind-Spot in Healthcare.* Berlin: Springer.

[B47] Winnicott D. W. (1954). Mind and its relation to the psyche-soma. *Br. J. Med. Psychol.* 27 201–209. 10.1111/j.2044-8341.1954.tb00864.x 13199220

[B48] Winnicott D. W. (1965). Communicating and not communicating leading to a certain opposites. in *The Maturational Process and the Facilitating Environment.* New York, NY: International University Press, 179–192.

[B49] Winnicott D. W. (1971). *Playing and Reality.* London: Burns & Oates.

[B50] Winnicott D. W. (2014). *Through Pediatrics to Psychoanalysis: Collected Papers.* Abingdon: Routledge.

[B51] Yalom I. D. (1980). Existential psychotherapy. 1980. New York, NY: Basic Books.

[B52] Yalom I. D. (2012). *Love’s Executioner: & Other Tales of Psychotherapy.* New York, NY: Basic Books.

